# RNA-seq identifies a diminished differentiation gene signature in primary monolayer keratinocytes grown from lesional and uninvolved psoriatic skin

**DOI:** 10.1038/s41598-017-18404-9

**Published:** 2017-12-22

**Authors:** William R. Swindell, Mrinal K. Sarkar, Yun Liang, Xianying Xing, Jaymie Baliwag, James T. Elder, Andrew Johnston, Nicole L. Ward, Johann E. Gudjonsson

**Affiliations:** 1 Ohio University, Heritage College of Osteopathic Medicine, Athens, OH 45701 USA; 2 University of Michigan, Department of Dermatology, Ann Arbor, MI 48109–2200 USA; 3 Department of Dermatology, Case Western Reserve University, 10900 Euclid Ave, Cleveland, OH 44106 USA; 4 The Murdough Family Center for Psoriasis, Case Western Reserve University, Cleveland, OH USA

## Abstract

Keratinocyte (KC) hyper-proliferation and epidermal thickening are characteristic features of psoriasis lesions, but the specific contributions of KCs to plaque formation are not fully understood. This study used RNA-seq to investigate the transcriptome of primary monolayer KC cultures grown from lesional (PP) and non-lesional (PN) biopsies of psoriasis patients and control subjects (NN). Whole skin biopsies from the same subjects were evaluated concurrently. RNA-seq analysis of whole skin identified a larger number of psoriasis-increased differentially expressed genes (DEGs), but analysis of KC cultures identified more PP- and PN-decreased DEGs. These latter DEG sets overlapped more strongly with genes near loci identified by psoriasis genome-wide association studies and were enriched for genes associated with epidermal differentiation. Consistent with this, the frequency of AP-1 motifs was elevated in regions upstream of PN-KC-decreased DEGs. A subset of these genes belonged to the same co-expression module, mapped to the epidermal differentiation complex, and exhibited differentiation-dependent expression. These findings demonstrate a decreased differentiation gene signature in PP/PN-KCs that had not been identified by pre-genomic studies of patient-derived monolayers. This may reflect intrinsic defects limiting psoriatic KC differentiation capacity, which may contribute to compromised barrier function in normal-appearing uninvolved psoriatic skin.

## Introduction

Psoriasis plaques develop from abnormal interactions between epidermal KCs and infiltrating immune cells, leading to KC hyper-proliferation with epidermal thickening^1,2^. The role of KCs in normal skin homeostasis and plaque formation is complex and multifaceted^3,4^. KCs generate chemokines and cytokines such as IL-1b, IL-6 and TNF, and also produce defensive antimicrobial factors including β-defensins, LL37 and S100 proteins^5,6^. Such KC-derived factors amplify inflammatory cascades and the cytokine network that underlies psoriasis plaque formation^7^. KCs also have a critical structural function in epidermal barrier formation, which requires proper execution of early-to-late KC differentiation stages, leading to an intact stratum corneum with abundant keratin and lipid matrix^4^. This process is altered in psoriasis lesions, however, as evidenced by dysregulated expression of suprabasal differentiation markers (e.g., KRT1 and KRT10) and premature formation of a thin cornified envelope with abnormally increased involucin abundance^8–10^. It remains unclear whether this unique phenotype is due to intrinsic KC defects or is instead secondary to inflammatory cascades initiated by other cell types (e.g., T cells, dendritic cells, neutrophils)^6,11^.

Transcriptome studies of full-thickness psoriatic skin can in principle provide insights for understanding how KCs from psoriatic skin may differ from those in uninvolved or normal skin^12–14^. These studies have identified thousands of differentially expressed genes (DEGs) from the comparison of lesional psoriatic skin (PP) to either non-lesional skin from psoriasis patients (PN) or normal skin from control subjects (NN). Comparisons between PN and NN skin have also identified DEGs, although typically many fewer DEGs have been identified from this comparison (relative to the PP vs. NN or PP vs. PN comparisons)^15^. It appears that at least some changes in gene expression identified from these studies can be traced to the epidermis or altered transcriptional activity of psoriatic KCs^13^. This conclusion has often been difficult to substantiate, however, primarily because epidermal thickness is greatly increased in lesional compared to non-lesional skin, which may lead to increased expression of KC-expressed genes in the absence of transcriptional up-regulation on a per-cell basis^13^. A second challenge has been that KCs within full-thickness biopsies remain embedded in their native multicellular environment, which includes influences of other cell types and factors released by those cell types (e.g., fibroblasts, immune cells, growth factors, cytokines, etc.)^1,5^. This permits identification of DEGs within a less disturbed *in vivo* context, but it is difficult to determine whether such DEGs stem from intrinsic KC defects or instead represent altered responses of KCs to the psoriatic skin microenvironment.

To identify intrinsic psoriatic KC defects, earlier studies have evaluated their phenotype in cell culture or transplant systems in which KCs are extracted from intact human skin^16–20^. This simplifies the extracellular environment to attenuate the influence of exogenous factors or other cell types, thereby facilitating comparisons among samples in a “common garden” context. One approach has been to compare *in vitro* phenotypes of PP/PN-KCs and NN-KCs in the setting of KC monolayer cultures grown from skin punch biopsies^21^. Compared to KCs from intact skin, it is expected that KCs grown as monolayers in culture will have altered proliferation and differentiation status, with the balance between proliferation and differentiation sensitive to cell culture conditions such as extracellular calcium levels^22^. Nonetheless, such studies have shown that PP-KCs and PN-KCs proliferate at roughly the same rate in monolayer cultures, despite drastically different rates of proliferation *in vivo* ^16,20^. PP-KCs were in fact shown to initiate proliferation more slowly than PN-KCs in primary cultures, with no observed difference in proliferation rates in serially passaged KC cultures^19^. Using the same approach, multiple studies were able to demonstrate elevated rates of DNA synthesis in both PP-KCs and PN-KCs as compared to NN-KCs^16,20^. This appeared to support the idea that psoriatic KCs (PP and PN) possess intrinsic properties differing from NN-KCs, while further providing proof-of-principle to demonstrate the utility of patient-derived KC monolayers as a model system for investigating this possibility^16,20^. These earlier studies, however, were carried out in the pre-genomic era and thus tools now available for large-scale transcriptome analysis could not be applied.

This study used RNA-seq to evaluate gene expression in primary confluent KC monolayer cultures (0.1 mM calcium) grown from full-thickness punch biopsies from psoriasis patients and control subjects (PP, PN and NN samples). We additionally used RNA-seq to concurrently evaluate gene expression in full-thickness skin biopsies from the same subjects. Psoriasis DEGs are identified based upon analysis of both sample types (monolayer KCs and full-thickness skin sections), and we compare DEGs identified from both analyses to identify points of correspondence and differences. Our analyses identify DEGs similarly altered in both sample types, which can be unambiguously assigned to KCs and likely represent instances of KC-specific transcriptional dysregulation. We also identify psoriasis DEGs uniquely altered in KC monolayers and not similarly altered in full-thickness skin sections. These novel psoriasis DEGs may arise from intrinsic alterations of psoriatic KCs impacting their *in vitro* expression profile.

## Results

### RNA-seq analysis of primary confluent monolayer KC cultures and whole skin biopsies from psoriasis patients and control subjects

Primary monolayer KC cultures were established using punch biopsies from lesional involved skin of psoriasis patients (PP), uninvolved non-lesional skin of psoriasis patients (PN), and normal skin from control subjects (NN) (*n* = 4 patients; *n* = 4 controls). All biopsies were processed in the same manner with cells harvested after reaching confluence. RNA was then extracted from KCs and a separate extraction was performed using the original skin biopsy from each subject (*n* = 24 samples total; 12 KC and 12 whole skin). We then used RNA-seq to generate an average of 27.6 million quality-filtered reads per sample, with at least 94% of the filtered reads successfully mapped to the human genome for each sample (Supplementary Figure S1).

An initial cluster analysis grouped together samples from the same subjects, suggesting that inter-individual variation had stronger influence than biopsy type (KC/skin) or disease status (PP/PN/NN) (Supplementary Figure S2A). Principal component analyses also showed that KC or skin samples from the same individual mapped to similar locations (Supplementary Figure S2C–F) and likelihood ratio tests (LRTs) confirmed that, for many genes, inter-individual differences contributed the most to variation among the 24 samples (Supplementary Figure S2G). Nonetheless, expression profiles of some NN-KC and NN-Skin samples differed from the psoriasis patient samples (Supplementary Figure S2C–F). We thus repeated the cluster analysis using expression values adjusted to remove the effects of patient/subject (i.e., residual analysis), which showed that NN-KC and NN-Skin samples grouped together and apart from the psoriasis patient samples (Supplementary Figure S2B).

### Transcriptome differences between psoriatic and normal KCs are larger than those in whole skin and overlap more with genes near disease-associated SNPs

**Figure 1:**
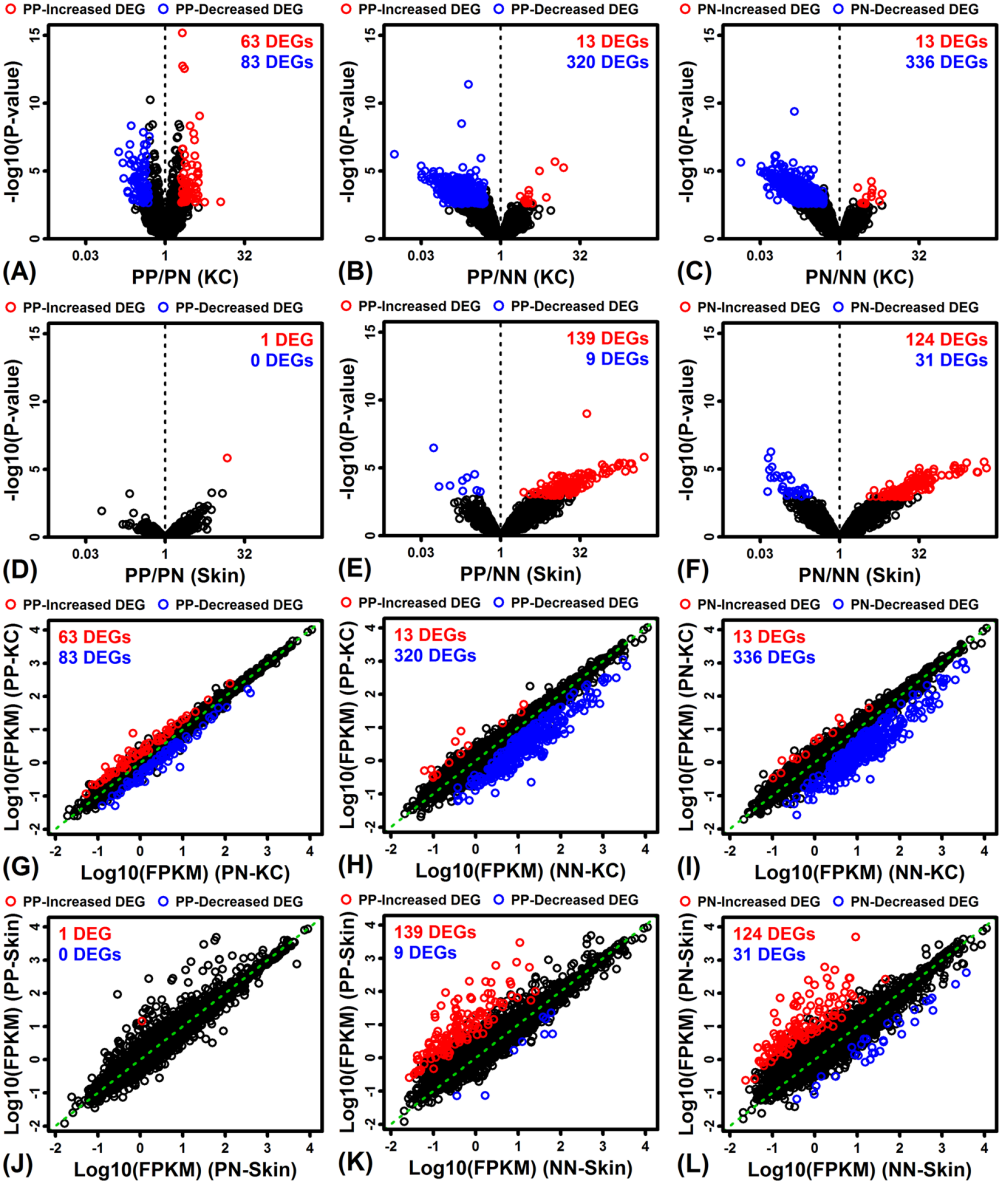
Differential expression results (PP vs. PN, PP vs. NN, PN vs. NN). Volcano plots highlighting DEGs identified with respect to the three comparisons for (**A**–**C**) KC and (**D**–**F**) full-thickness skin biopsies (FDR < 0.10 with FC > 2.0 or FC < 0.50). (**G**–**L**) Scatterplot comparison of average FPKM in treatments compared by each differential expression analysis (dashed green line: equal average FPKM in both treatments).

We identified differentially expressed genes (DEGs) based upon 3 comparisons (PP vs. PN, PP vs. NN, PN vs. NN) and repeated the analysis for KC and skin samples (Fig. 1). Surprisingly, for all 3 comparisons, the number of DEGs identified in KCs was larger than the number identified in whole skin biopsies (Fig. 1A–C vs. 1D–F). The strongest difference was observed for genes with decreased expression in psoriatic samples. For instance, compared to NN-KCs, more than 300 DEGs were decreased in PP-KCs and PN-KCs (FDR < 0.10 and FC < 0.50) (Fig. 1). However, compared to NN-Skin, only 9 and 31 DEGs were decreased in PP-Skin and PN-Skin, respectively (Fig. 1).

**Figure 2:**
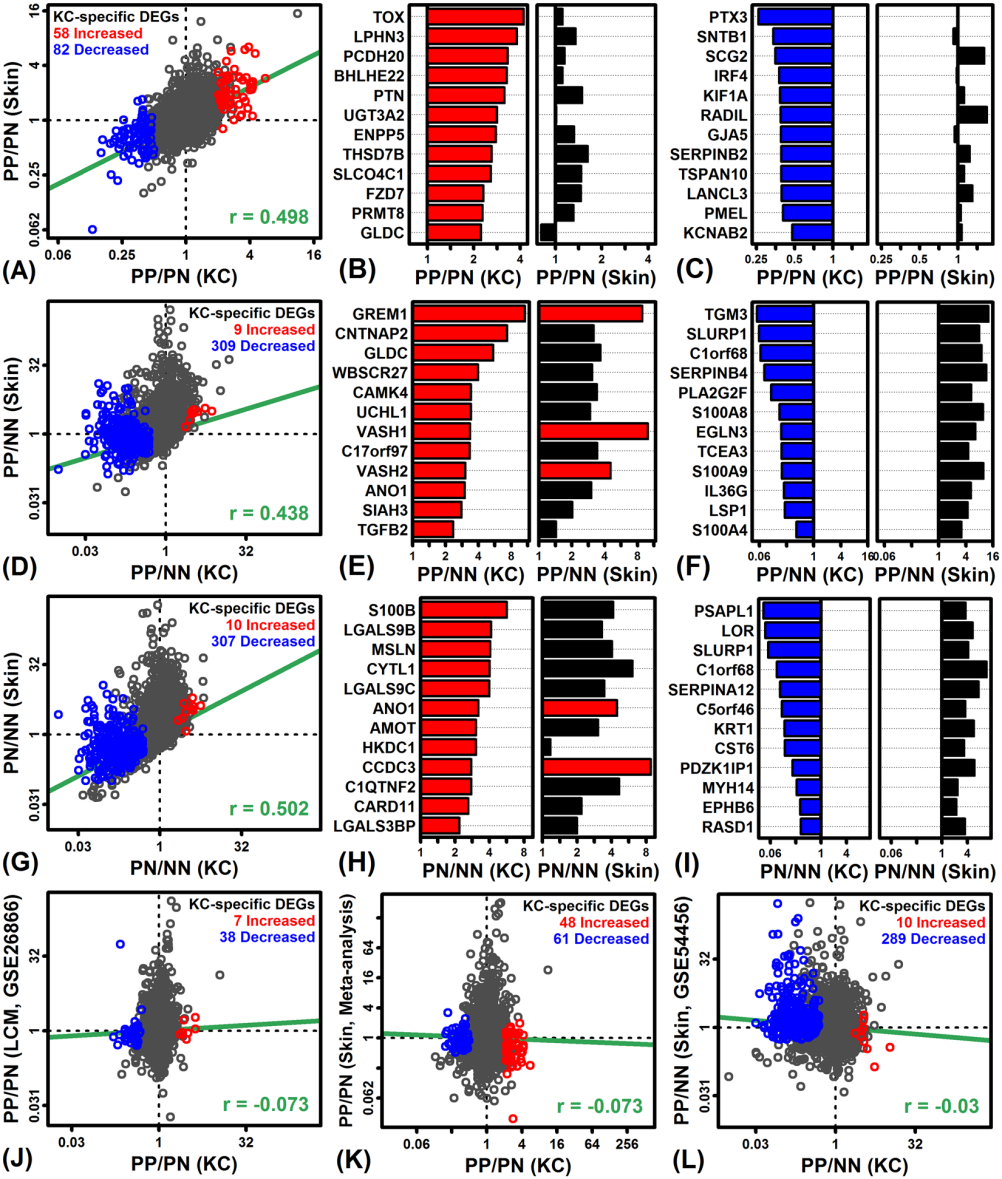
Genes with decreased expression in PP-KCs and PN-KCs are not similarly altered in whole skin biopsy comparisons. (**A**,**D**,**G**) Comparison of FC estimates from whole skin (vertical axis) and KC comparisons (horizontal axis). (**B**,**E**,**H**) PP/PN-KC-increased genes with the least similar expression changes compared to whole skin (red: FDR < 0.10, FC > 1.50). (**C**,**F**,**I**) PP/PN-KC-decreased genes with the least similar expression changes compared to whole skin (blue: FDR < 0.10, FC < 0.67). (**J**,**K**,**L**) Comparison of FC estimates from KCs to those from (**J**) LCM-dissected epidermis (GSE26866), (**K**) an RNA-seq study of 44 patients (GSE41745, GSE54456/GSE63979, GSE66511), and (**L**) an RNA-seq study of 174 subjects (92 PP, 82 NN; GSE54456).

Overall, FC estimates showed modest correspondence between monolayer KCs and whole skin (*r*_s_ ≥ 0.438; Fig. 2A,D,G). Most genes elevated in PP- or PN-KCs were similarly elevated in PP- or PN-Skin, as compared to NN-KCs and NN-Skin, respectively (Fig. 2B,E,H). However, many genes decreased in PP-KCs and PN-KCs (compared to NN-KCs) were not similarly decreased in the comparison between PP/PN-Skin and NN-Skin (Fig. 2A–I). Such genes included *SERPINB4*, *S100A8*, *S100A9*, *IL36G*, *LOR* and *KRT* (Fig. 2C,F and I). The KC-Skin correspondence was not improved by comparing our KC results to those from a prior microarray analysis of LCM-dissected psoriatic epidermis (Fig. 2J) or RNA-seq studies of whole skin with a larger number of patient samples (Fig. 2K and L). Analysis of KC monolayer cultures thus identified gene expression differences not discernable from either whole skin analysis or LCM-dissected samples.

**Figure 3:**
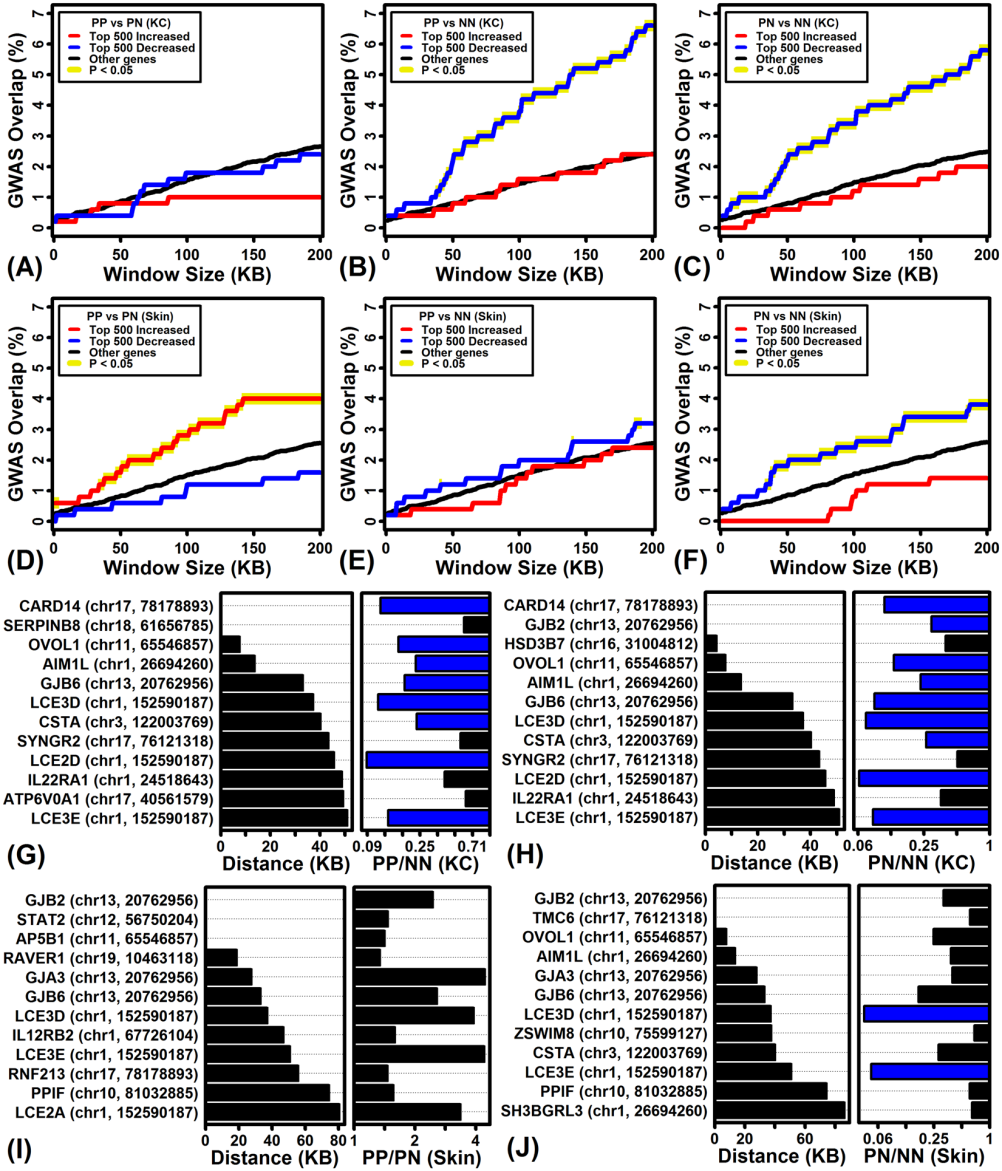
Genes with decreased expression in PP-KCs and PN-KCs overlap significantly with genes near SNPs identified by psoriasis GWA studies. (**A**–**F**) Genes altered in psoriatic KCs (**A**–**C**) or whole skin (**D**–**F**) and their overlap with genes near SNPs from psoriasis GWA studies. The 500 most strongly altered genes were examined in each analysis and percent overlap with genes at varying distances from psoriasis-associated SNPs was evaluated (yellow: P < 0.05, Fisher’s Exact Test). (**G**–**J**) Genes most strongly altered in psoriasis KCs or skin (top 500) that are closest to a psoriasis-associated SNP (left margin). The estimated FC is shown for each gene (red: FDR < 0.10, FC > 2.0; blue: FDR < 0.10, FC < 0.50).

We next compared genes most strongly altered in our comparisons to genes at varying distances from disease-associated SNPs identified by genome-wide association (GWA) studies of psoriasis (Fig. 3)^23^. Genes with elevated expression in PP-Skin and decreased expression in PN-Skin overlapped significantly with genes near disease-associated SNPs (Fig. 3D,E). Overlap was much stronger, however, with respect to genes with decreased expression in PP-KCs and PN-KCs compared to NN-KCs (50–200 KB; Fig. 3B,C). Genes decreased in PP-KCs and PN-KCs with closest proximity to disease-associated SNPs were often located on chromosomes 1 or 17 and included *CARD14*, *LCE2D*, *LCE3E* and *LCE3D* (Fig. 3G,H).

### Psoriatic KCs exhibit increased expression of pro-angiogenesis genes and decreased expression of early and late differentiation genes

DEGs with elevated expression in PP-KCs compared to PN-KCs or NN-KCs were significantly enriched with genes associated with neovascularization, angiogenesis, blood vessel morphogenesis (Supplementary Figures S3 and S4). The strongest trend we observed, however, was reduced expression of genes associated with epidermal development and differentiation (PP-KCs and PN-KCs compared to NN-KCs; Supplementary Figures S4 and S5). This loss of differentiation-associated gene expression appeared to contribute to differences noted above in comparisons to whole skin biopsies (Fig. 2). Several differentiation-associated genes, for instance, were repressed in psoriatic KCs but not whole skin biopsies (e.g., *TGM3*, *LOR*, *KRT1*; Fig. 2F,I).

**Figure 4:**
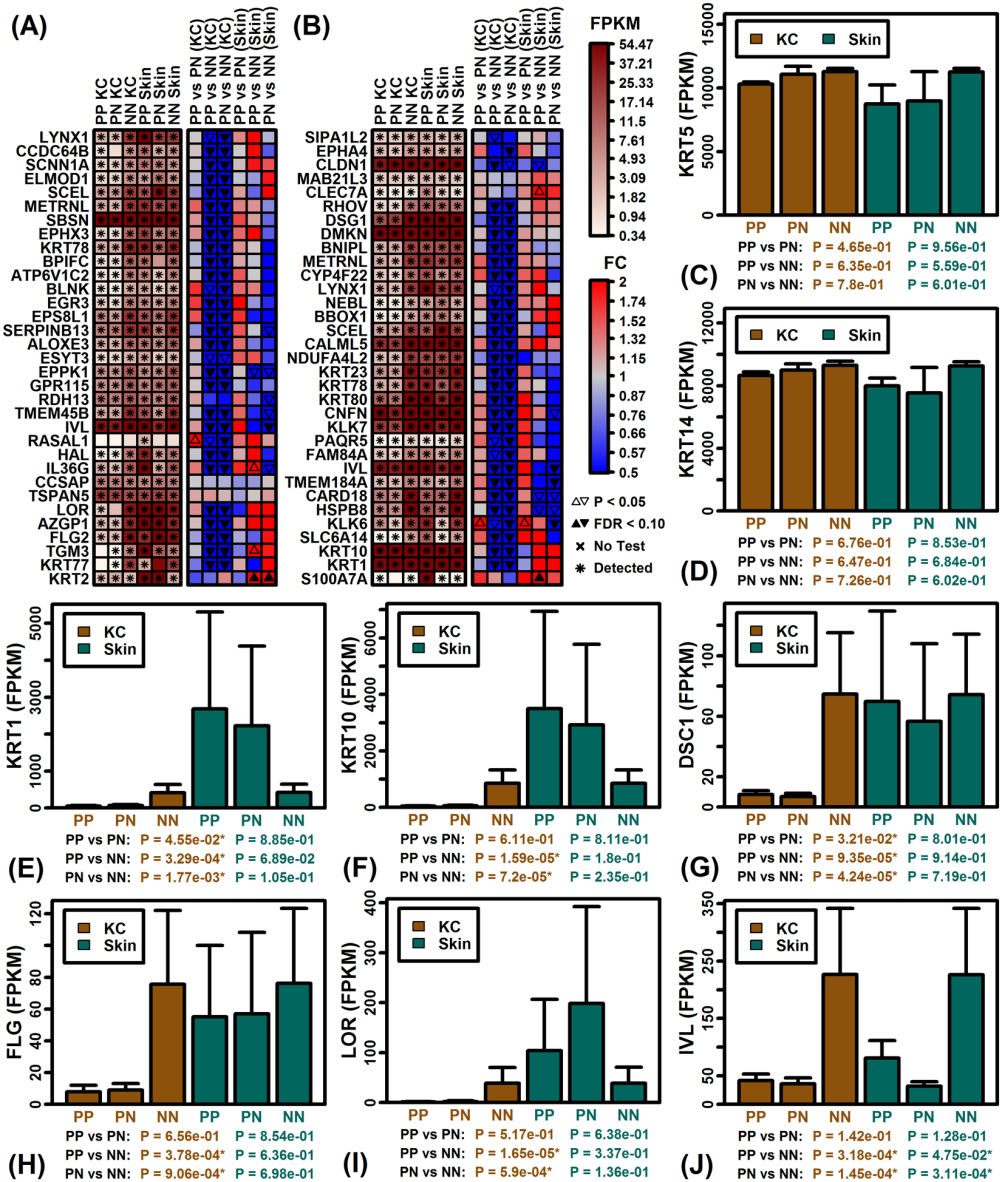
Early and late differentiation gene expression is reduced in PP- and PN-KCs compared to NN-KCs. (**A**,**B**) FPKM and FC estimates for genes with (**A**) increased expression in LCM-dissected suprabasal epidermis compared to basal epidermis (GSE42114) and (**B**) increased expression in KCs undergoing differentiation (devitalized dermis model, GSE52651). (**C**,**D**) Average FPKM of basal keratin genes *KRT5* and *KRT14*. (**E**–**G**) Average FPKM of early differentiation genes *KRT1*, *KRT10* and *DSC1*. (**H**–**J**) Average FPKM of late differentiation genes *FLG*, *LOR* and *IVL*.

Using data from a prior microarray study of LCM-dissected human skin^24^, we identified genes with elevated expression in suprabasal vs. basal epidermis, and found that nearly all of these were decreased in PP- and PN-KCs compared to NN-KCs (Fig. 4A). A similar pattern was observed among genes up-regulated in KCs differentiated within a devitalized dermis model of epidermal regeneration (Fig. 4B)^25^. Neither of these trends, however, could be discerned from analysis of full-thickness skin biopsies. Consistent with these results, PN- and PP-KCs showed decreased expression of early (*KRT1*, *KRT10*, *DSC1*) and late (*FLG*, *LOR*, *IVL*) differentiation genes (Fig. 4E–J). Basal keratins *KRT5* and *KRT14* did not show altered expression in monolayer KCs or full-thickness biopsies (Fig. 4C,D). Markers of hyperproliferation, such as *KRT72* (*KRT6*) and *KRT16*, were increased slightly in PP versus PN skin but not PP-KCs versus PN-KCs; interestingly, however, *KRT16* expression was elevated in NN-KCs compared to PP- and PN-KCs (Supplementary Figure S6).

### Psoriatic monolayer KCs exhibit shifts in cytokine gene expression distinct from those in full-thickness skin biopsies

*IL36G* encodes an interleukin 1 family cytokine and has been identified as a robust biomarker of lesional psoriatic skin, with one meta-analysis study showing increased expression of *IL36G* in 100% of lesional skin samples from 237 psoriasis patients (compared to matched non-lesional PN skin from the same patients)^26^. Remarkably, *IL36G* expression was decreased in PP- and PN-KCs compared to NN-KCs, although as expected expression was much higher in PP-skin compared to PN- and NN-skin (Supplementary Figure S7). We additionally observed decreased expression of *IL36RN*, *IL20RA*, *IL22RA1*, *TNFAIP8L3* in PP- and PN-KCs without similar expression shifts in full-thickness skin biopsies (Supplementary Figure S7). We did not detect *IL17A* or *TNF* expression in KC or full-thickness skin samples, and there was no indication that genes decreased in PP- or PN-KCs are repressed by these cytokines (Supplementary Figure S7).

### Identification of an AP-1 motif enriched in regions upstream of PN-KC-decreased DEGs and within open chromatin regions near differentiation-associated genes

The magnitude of PN vs. NN differences has been limited in prior transcriptome studies of full-thickness skin biopsies^15^. It was therefore surprising to find strong differences between PN-KCs and NN-KCs, with identification of 336 PN-KC-decreased DEGs (Fig. 1C). We evaluated whether genomic sequences 5000 BP upstream of the 336 PN-KC-decreased DEGs were enriched for motifs known to participate in sequence-specific interactions with transcription factors (TFs) or unconventional DNA-binding proteins (uDBPs). Of 2935 motifs screened^26^, 277 were significantly enriched in regions upstream of the 336 PN-KC-decreased DEGs (FDR < 0.10). A significant proportion of the 277 motifs were associated with Fos- and Jun-related TFs from the bHLH/bZIP class and basic superfamily (Supplementary Figure S8).

Only 34 of the 277 significant motifs interacted with TFs or uDBPs with decreased expression in PN-KCs compared to NN-KCs (FDR < 0.10). These 34 motifs included one recognized by KLF4 and several others associated with AP-1 (Supplementary Figure S8E and F). Consistent with this, several genes encoding Jun- and Fos-related TFs trended towards decreased expression in PN-KCs compared to NN-KCs, although only *FOSL2* and *JUNB* met criteria as DEGs with FDR < 0.10 and FC < 0.50 (Supplementary Figure S8D). We identified one motif (5-TGACTCA/TGAGTCA-3) interacting with Fra-2 (FOSL2) that was present in upstream regions of several genes known to influence KC differentiation, including *DSC2*, *IVL*, *FLG*, *KLF4*, *CASP14*, and *LOR* (Supplementary Figure S9). Several occurrences of this motif were in open chromatin regions defined by ENCODE studies of NHEK cells (Supplementary Figure S9B and C)^27^.

### A module of 36 co-expressed genes (ACER1-36) with differentiation-dependent expression is down-regulated in KCs from lesional and uninvolved psoriatic skin

**Figure 5:**
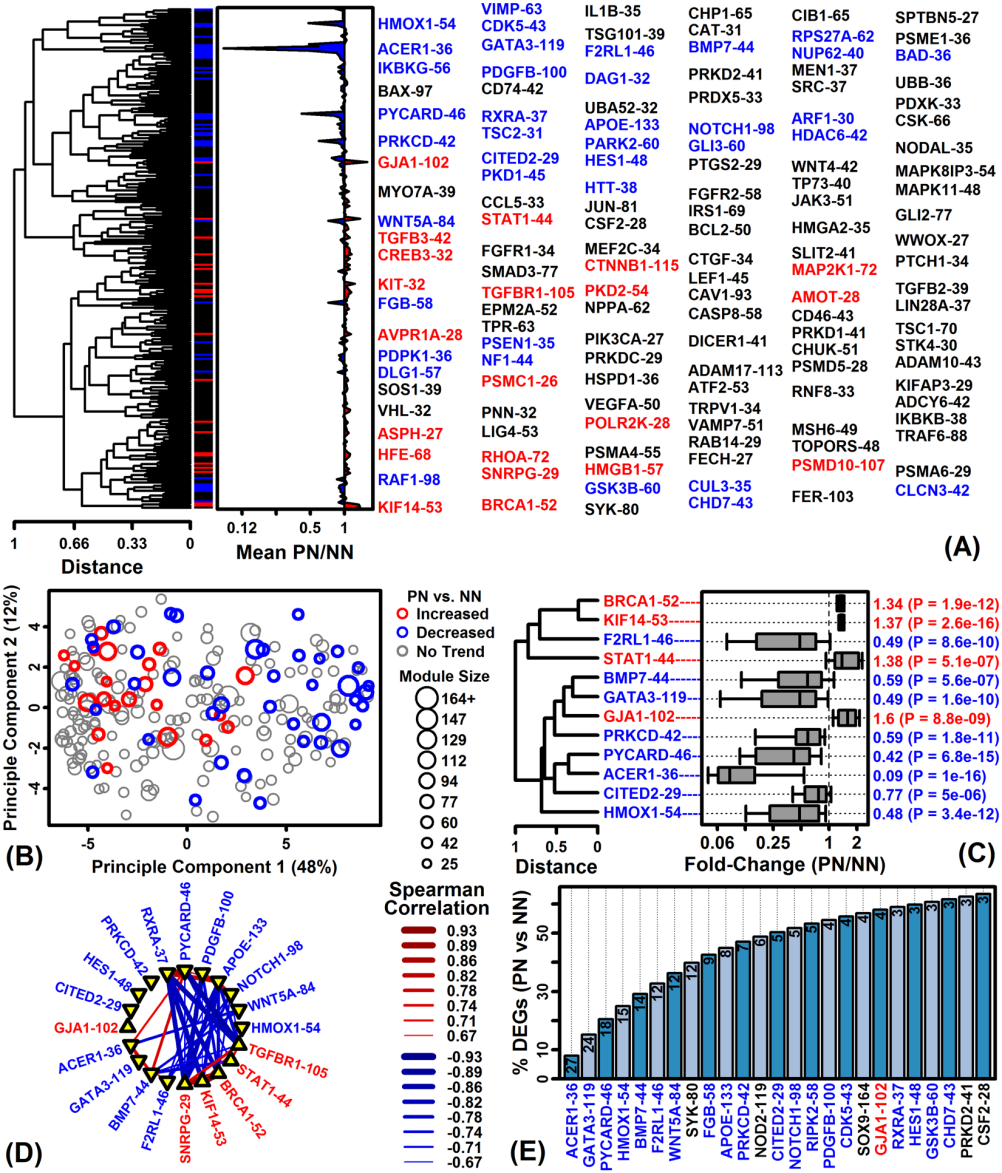
Identification of differentially expressed modules (DEMs) from the comparison of PN-KCs to NN-KCs. (**A**) Cluster analysis of 239 co-expression module medoids (Euclidean distance; horizontal axis: average FC among module genes). Module IDs are shown (right margin; red: PN-KC-increased DEMs; blue: PN-KC-decreased DEMs). (**B**) Module medoids plotted with respect to principal component axes (red: PN-KC-increased DEMs; blue: PN-KC-decreased DEMs). (**C**) Cluster analysis of top 12 DEM medoids (Euclidean distance; grey boxes: middle 50% of FC estimates; whiskers: middle 80% of FC estimates). (**D**) Spearman rank correlations among medoids (top 20 DEMs). (**E**) Cumulative percentage of the 336 PN-KC-decreased DEGs accounted for by modules (black font: number of PN-KC-decreased DEGs in each module).

We used hierarchical clustering to group genes into 239 modules (≥25 genes per module) based upon co-expression patterns across an independent dataset (i.e., 82 normal human skin samples from control subjects; Fig. 5A)^14^. Of 239 modules, we identified 61 differentially expressed modules (DEMs) with member genes biased towards PN-KC-increased (21 DEMs) or –decreased (40 DEMs) expression. Increased and decreased DEM medoids were often negatively correlated (Fig. 5C,D) and approximately 20% of PN-KC-decreased DEGs could be accounted for by only 3 modules (ACER1-36, GATA3-119, PYCARD-46) (Fig. 5E).

**Figure 6:**
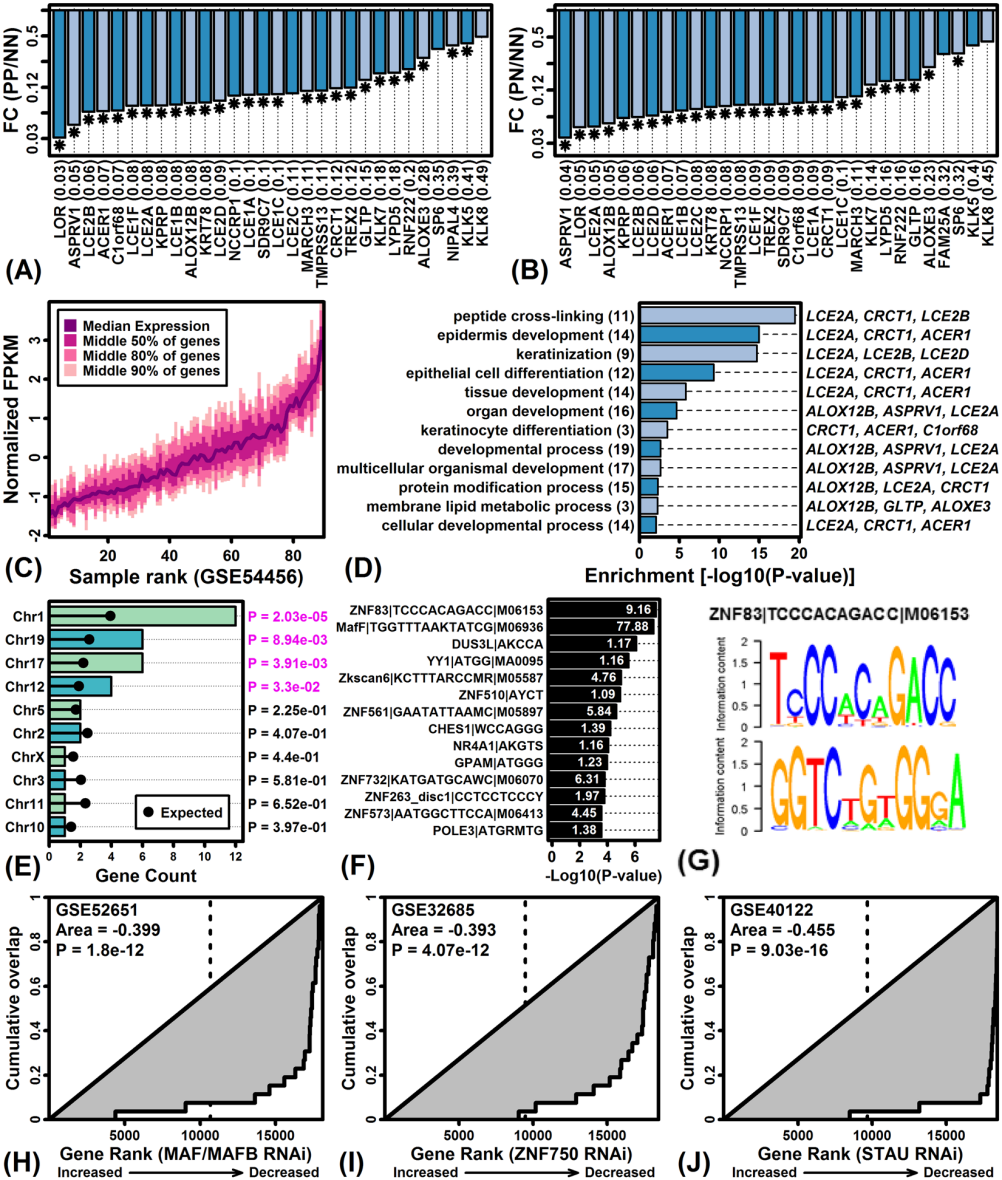
The ACER1-36 module is decreased in PP- and PN-KCs and exhibits differentiation-dependent expression. (**A**,**B**) ACER1-36 genes ranked according to their expression decrease (PP/PN-KCs, bottom margin: FC). (**C**) ACER1-36 expression in NN skin (GSE54456). (**D**) GO BP terms enriched a mong ACER1-36 genes (left margin parentheses: number of genes per term). (**E**) Chromosomes associated with AVER1-36 genes (right margin: Fisher’s Exact Test p-values). (**F**) Motifs enriched in regions 5000 bp upstream of ACER1-36 genes (white font: observed/expected motif frequency). (**G**) ZNF83 sequence logo. (**H**–**J**) ACER1-6 genes show decreased expression in KCs following RNAi knockdown of (**H**) MAF and MAFB, (**I**) ZNF750, and (**J**) STAU. Genes are ranked according to their RNAi expression change and cumulative overlap with ACER1-36 genes is shown.

ACER1-36 included genes with the strongest and most consistent expression differences between PN-KCs versus NN-KCs (Fig. 5A,C; Fig. 6A). 27 of 36 genes were decreased significantly in PN-KCs compared to NN-KCs (FDR < 0.10 with FC < 0.50), and most genes were similarly decreased in PP-KCs compared to NN-KCs (Fig. 6B). A significant fraction of the 36 genes (12/36) were located on chromosome 1 within the 1q21 segment that includes the epidermal differentiation complex (EDC) (Fig. 6E). Consistent with this, ACER1-36 was significantly enriched for genes associated with epidermis development, keratinization, and cell differentiation (Fig. 6D). Nearly all 36 genes were repressed by treatments that block normal keratinocyte differentiation, such as RNAi knockdown of *MAF*/*MAFB*, *ZNF750* and *STAU* (Fig. 6H–J). Analysis of 5000 BP regions upstream of ACER1-36 identified enrichment for DNA motifs recognized by zinc finger protein transcription factors as well as a motif that interacts with MAF bZIP transcription factor F (*MAFF*) (Fig. 6F,G).

## Discussion

The classic conception of psoriasis as a cutaneous disease has evolved towards a more nuanced understanding of psoriasis as a hybrid immune-cutaneous condition resulting from abnormal interactions between KCs and activated immune cells^1,2^. The possibility that intrinsic KC defects drive plaque formation, at least in part, has been frequently debated but remains unresolved^3,6,11^. This study used primary monolayer cultures as a model system to compare KCs grown from lesional (PP), uninvolved (PN), and normal human skin (NN). Altered expression of differentiation-associated genes was the strongest transcriptomic feature distinguishing PP/PN-KCs from NN-KCs, with PP/PN-KCs exhibiting decreased expression of early/late differentiation markers (*KRT1*, *KRT10*, *FLG*, *LOR*) and differentiation mediators (*CASP14*, *ACER1*). A subset of PP/PN-KC-repressed genes was associated with AP-1 binding sites and belonged to a single co-expression module (ACER1-36) mapping to the EDC. These results highlight differentiation as a key dysregulated process that may drive other aspects of the *in vitro* PP/PN phenotype (e.g., increased DNA synthesis and cell proliferation). Our findings are additionally consistent with the idea that PN-KCs have intrinsic defects limiting their differentiation capacity, which supports a model of pathogenesis in which plaque formation results at least in part from a primary cutaneous defect.

Transcriptome studies of full-thickness lesional and uninvolved psoriatic skin have been advanced to discover unique cutaneous features of psoriatic skin, which may allow for the identification of genes with KC-specific changes in transcriptional activity^12–14^. Transcriptome findings from this *in vitro* study, however, agreed only partially with results from full-thickness skin biopsies (Fig. 2). For example, several genes known to be robustly elevated in full-thickness sections of PP skin were in fact down-regulated in PP-KCs compared to NN-KCs (e.g., *S100A8*, *S100A9*, *IL36G*; Fig. 2). This seems paradoxical, and may reflect differential response patterns of such genes in the context of cell culture as compared to intact skin^22,28^. Alternatively, such genes may be predominantly KC-expressed and thus tend to show elevated expression in PP skin biopsies, due to expansion of the epidermal compartment^13^. Additional examples include genes within the EDC previously identified as up-regulated in PN skin compared to NN skin (e.g., *SPRR2B*, *SPRR2G*, *SPRR3*, *LCED3*)^15^. These findings suggest that analysis of full-thickness skin biopsies may obscure functionally important expression declines in PP- and PN-KCs, which can in contrast be detected from *in vitro* analysis of patient-derived cells (Fig. 2). This may explain why genes with decreased expression in full-thickness psoriatic skin biopsies included a lower proportion of genes near loci identified by psoriasis GWA studies^23^, whereas such overlap was stronger among genes decreased in PP/PN-KCs compared to NN-KCs (Fig. 3).

Uninvolved skin from psoriatic patients appears macroscopically normal, although previous studies have identified impaired barrier recovery along with decreased abundance of late differentiation markers, such as the cornified envelope proteins filaggrin (*FLG*) and loricrin (*LOR*)^29^. In agreement with these findings, expression of *FLG* and *LOR* was decreased in PN-KCs compared to NN-KCs (Fig. 4). We additionally identified decreased expression of involucrin (*IVL*) in PN-KCs (Fig. 4), which was not previously observed in analyses of full-thickness skin sections^29^. It has been proposed that decreased expression of *FLG* and *LOR* in uninvolved psoriatic skin is secondary to elevated TNF-α expression in PN compared to NN skin^29^. We did not detect TNF expression in our samples and there was no indication that TNF represses expression of PN-KC-decreased DEGs (Supplementary Figure S7). In our experiments, KCs harvested from skin biopsies underwent repeated wash steps in monolayer cultures, which we expected to equalize the extracellular environment and cytokine concentrations of PN-KCs and NN-KCs. It is possible, however, that elevated TNF-α in PN skin alters the methylation status of promoter elements associated with *FLG*, *LOR*, and *IVL*, which may then be inherited in subsequent cell divisions to explain loss of *FLG*, *LOR*, and *IVL* expression in PN-KCs^30^. Alternatively, psoriatic KCs may harbor disease-associated genetic variants leading to decreased expression of genes required to execute the full differentiation program, including the EDC-associated ACER1-36 module identified in this study (Figs 5 and 6).

Transcription factors (TFs) regulate normal epidermal differentiation and genetic variants that alter TF activity may contribute to impaired KC differentiation of psoriatic skin^31,32^. Consistent with this, expression of *KLF4* and *KLF4*-regulated genes was decreased in PN-KCs compared to NN-KCs. PN-KCs also showed decreased expression of genes encoding AP-1 transcription factors (*FOSL2* and *JUNB*), and motifs associated with Jun- and Fos-related TFs were enriched in regions upstream of the 336 PN-KC-decreased DEGs. This appears consistent with prior work suggesting that decreased AP-1 activity in psoriatic KCs may precede plaque formation^33^. In mice, epidermal deletion of *JUNB* and *JUN* was shown to promote a psoriasiform skin phenotype with arthritic lesion formation^33^. A recent genomic analysis also demonstrated that risk alleles at enhancer-associated non-coding psoriasis susceptibility loci frequently disrupt AP-1 binding sites^26^. These findings and our current data suggest that loss of AP-1 activity or DNA binding may impair differentiation of psoriatic KCs, and such effects may be synergistic with decreased *KLF4* expression. Within the *in vivo* setting, this may have an initiating effect on plaque development by weakening the PN epidermal barrier, thereby lowering the threshold for triggering inflammatory responses^3,26^.

It is important to note that our study was performed using KCs cultured under relatively low calcium concentrations (0.1 mM), which may have favored a proliferative phenotype and inhibited KC differentiation regardless of the donor’s disease status^34,35^. While we expect some differentiation markers to be expressed at this calcium concentration^34,35^, experiments performed with higher concentrations (e.g., 1.2–2.4 mM) would have provoked further cell-to-cell contact, culture stratification and differentiation. Potentially, higher calcium concentrations may have widened PP/PN versus NN differences with respect to the expression of differentiation-associated genes, although further studies would be needed to evaluate this possibility, with PP/PN- and NN-derived KCs cultured under varying calcium concentration levels. We also note that our analyses have identified differences in mRNA expression, whereas further characterization of KC differentiation status would require assays for key differentiation marker proteins (e.g., KRT1, KRT5, KRT10, KRT14, FLG, LOR and IVL). Finally, in this study we concurrently analyzed expression profiles of KC monolayer cultures and whole skin biopsies from the same patients. This allowed us to compare KC and whole skin PP/PN versus NN expression signatures while controlling for patient heterogeneity^36^. However, an alternative approach for future work may be to perform similar comparisons using epidermal isolates rather than full-thickness biopsies (e.g., ammonium thiocyanate incubation or laser capture microdissection)^24,37^. This would limit the influence of non-KC cell types and may yield a PP/PN versus NN signature more correspondent with that observed in monolayer cultures.

This study extends previous transcriptome analyses to include RNA-seq analysis of patient-derived KC monolayer cultures, which had not yet been investigated using the modern toolkit of sequencing and bioinformatic methods. Our transcriptomic findings uncover novel expression differences among PP, PN and NN skin samples that are not discerned from analysis of full-thickness skin sections. Such differences can be unambiguously localized to KCs and may reflect intrinsic disease-associated features of the psoriatic KC phenotype. Our results provide direction for future studies to further address longstanding questions regarding the contribution of KCs to plaque formation in psoriatic disease.

## Methods

### Ethics statement

Samples were obtained from volunteer patients with informed written consent in accordance with Declaration of Helsinki principles. All protocols were approved by an institutional review board (University of Michigan, Ann Arbor, MI, IRB No. HUM00037994).

### Patient samples

6 mm punch biopsies were obtained from 4 psoriasis patients (PP, PN) and 4 normal control subjects without psoriasis (NN). The average age of the psoriasis patients was 46.5 years (range: 42–53; 3 males, 1 female) and the average age of the controls was 51.2 years (range: 29–61; 2 males, 2 females). PP samples were obtained from the central region of an active psoriasis plaque located on the forearm, leg, abdomen or flank region. PN and NN samples were obtained from sun-protected skin of the buttock or upper thigh region. Prior to biopsy collection, psoriasis patients had discontinued systemic therapies for at least 2 weeks, and had not used topical treatment for at least 1 week. Biopsies were obtained following local lidocaine injection for anesthesia and split into two fractions. One fraction was flash frozen in liquid nitrogen and stored at −80 °C until further processing for RNA extraction. The second fraction was processed immediately to generate primary KC monolayer cultures.

### Keratinocyte monolayer cultures

Skin biopsies were washed in HBSS containing antibiotics to remove blood, fat was trimmed away, and the remaining tissue was rinsed in 70% ethanol before being incubated in 50U/ml dispase (Gibco) at 37 °C for 2 hours to release epidermis from dermis. Epidermal sheets were incubated in 0.05% Trypsin-EDTA (Invitrogen) for 15 min at 37 °C, after which a slurry of cells was apparent. Trypsin was neutralized by the addition of complete Medium 154 culture medium containing 1% HKGS, 0.1 mM Ca^2+^ and antibiotics (Invitrogen). Cells were passed through a 100 µm cell strainer (BD Biosciences), washed, suspended in fresh culture medium, and seeded into 6-well cell culture plates (BD Biosciences). Cells were left undisturbed for 6 days at 37 °C with 5% CO_2_. Thereafter medium was replenished every 2–3 days. Cells were harvested shortly after reaching confluence and further processed for RNA extraction.

### High throughput RNA sequencing

Total RNA was processed for high throughput sequencing using the Illumina TruSeq mRNA Sample Prep v2 kit (catalog no. RS-122-2001 and RS-122-2002). mRNA was generated by polyA purification using approximately 0.1–3.0 µg of total RNA per sample. Following fragmentation, mRNA was converted to cDNA using random primers and reverse transcriptase. Adaptor barcodes were added to permit multiple samples to be sequenced in each lane of a HiSeq flow cell (Illumina). Final cDNA libraries were purified and enriched by PCR (Kapa’s library quantification kit for Illumina Sequencing platforms; catalog no. KK4835; Kapa Biosystems, Wilmington MA). Quality assessment and cDNA quantification was performed using the Agilent TapeStation. Samples were clustered using Illumina’s automated clonal amplification system (cBot) and run with 6 samples per lane on a 50 cycle single end Illumina HiSeq 2000.

### Gene expression quantification

Sequencing generated an average of 28.0 million 50 bp reads per sample (*n* = 24 samples). These reads were further processed by Cutadapt and the FASTX-Toolkit to remove adaptor sequence and filter out low-quality reads, yielding an average of 27.6 million reads per sample (Supplementary Figure S1)^38,39^. FastQC was used to assess quality control parameters before and after read filtering^40^. The quality-filtered reads were then mapped to the human genome (hg19, UCSC) using tophat2 under default settings^41^, except only a single alignment was permitted per read and the coverage based junction search was disabled (settings: -g 1–no-coverage-search). Samtools was used to sort and index BAM alignment files and to calculate BAM file statistics^42^. HTSeq was used to tabulate the number of reads mapping to each genomic feature, with counts tabulated only for genes that completely overlapped a given feature, with at least some sequence uniquely mapping to the assigned feature and no other (-m intersection-strict)^43^. Cufflinks was then used to calculate FPKM estimates of gene expression along with associated confidence intervals^44^. RSeQC and RNA-SeQC were used to assess quality of sequence alignments (e.g., percentage of mapped and mapped intragenic reads, expression profiling efficiency)^45,46^.

### Differential expression analysis

Tests for differential expression were performed for protein-coding genes with detectable expression in at least 2 of 8 samples involved in a given comparison. For a gene to be considered as having detectable expression in a given sample, we required greater than 0.25 cpm (count per million mapped reads) with the lower limit of the FPKM 95% confidence interval greater than zero. Applying these criteria, we detected an average of 13660 protein-coding genes among the 24 samples (Supplementary Figure S1F). For genes with detectable expression in 2 of 8 samples involved in a given comparison, tests for differential expression were performed by fitting negative binomial generalized log-linear models to normalized gene counts, followed by likelihood ratio tests to assess statistical significance of expression differences (PP vs. PN, PP vs. NN, or PN vs. NN)^47^. Gene counts were normalized using the weighted trimmed mean of M-values method^48^, and negative binomial model dispersions were estimated using the Cox-Reid (CR)-adjusted likelihood approach^49^. For the PP vs. PN comparison, patient was included as an additional covariate in log-linear models to account for sample pairing, whereas the other comparisons (PP vs. NN, PN vs. NN) involved two independent groups without additional model covariates. Raw p-values from differential expression analyses were adjusted using the Benjamini-Hochberg method^50^.

### Identification of differentially expressed modules (DEMs)

A set of 13045 KC-expressed protein-coding genes was clustered with respect to expression patterns (FPKM) across 90 normal human skin biopsies from non-psoriatic control subjects^14^. The 13045 genes were each detected in at least 2 of the 8 samples for each of the 3 KC sample comparisons (i.e., PP vs. PN, PP vs. NN, PN vs. NN). Prior to clustering, expression of each gene was centered such that the average expression across samples was equal to zero. Expression values were then standardized to have the same mean and unit variance in all samples. The 13045 genes were clustered using average linkage hierarchical clustering and the Euclidean distance metric (R function: hclust). The resulting dendrogram was used to assign genes to each of 239 modules with a minimum module size of 25 genes (R package: dynamicTreeCut). Label assigned to each module were based upon the member gene most heavily annotated with Gene Ontology (GO) terms and the number of genes within the module. For example, the ACER1-36 module included 36 genes, of which *ACER1* was annotated with the largest number of GO terms.

Differentially expressed modules (DEMs) were identified based upon the comparison of expression in PN-KCs and NN-KCs. To identify DEMs, we evaluated whether genes within a module were more likely to be PN-KC-increased or PN-KC-decreased as compared to all other genes included in the cluster analysis (Wilcoxon rank sum test). The test was applied to each of the 239 modules and raw p-values were adjusted using the Benjamini-Hochberg method. Since modules included differing numbers of genes, this test was performed using only the 25 genes in each module that were closest to the module’s centroid (Euclidean distance), with the module’s centroid calculated by averaging expression of genes in each of the 90 samples used for cluster analysis. This ensured that DEMs were identified based only upon genes most representative of each module, and additionally equalized statistical power across modules for identification of DEMs. Overall, we identified 40 PN-decreased DEMs for which the median KC-PN/KC-NN FC was less than zero (FDR < 0.05), along with 21 PN-increased DEMs with median KC-PN/KC-NN FC greater than zero (FDR < 0.05).

### Comparison to genes identified by psoriasis GWA studies

A curated list of genome-wide significant risk variants from prior psoriasis GWA studies was obtained from an earlier publication^23^. Only studies testing for associations with psoriasis vulgaris (PsV) were included (i.e., studies testing for associations with psoriatic arthritis were excluded). The initial list included 129 PsV-associated loci from 67 independent regions^23^. This list was filtered to include only one locus from each independent region, with the most significant locus retained in those cases where the same region was associated with multiple PsV-associated loci. Protein-coding genes were then identified at varying distances from the 67 independent PsV-associated loci, and we evaluated overlap with respect to genes with expression most strongly altered in our experiments (Fisher’s Exact test; Supplementary Figure S3).

### DNA motif analysis

DEGs and DEMs were investigated to identify DNA motifs enriched in regions upstream of transcription start sites (TSS). A 5500 bp region was evaluated for DNA motif matches for each gene (5000 bp upstream of TSS, 500 bp downstream of TSS). A comprehensive set of 2935 motifs was included in the analysis, which had been assembled, filtered and annotated in a prior study^26^. The 2935 motifs were drawn from multiple source databases, including the human protein-DNA interaction database (hPDI), Jaspar, UniPROBE, TRANSFAC, and the ENCODE project^26^. For each DEG set or DEM, enrichment of motifs within 5500 bp TSS-proximal regions was evaluated using semiparametric generalized additive logistic models (GAM)^51^. The Benjamini-Hochberg method was used to correct raw p-values across the complete set of 2935 motifs included in the analysis^50^.

### Data Availability

 All raw and processed gene expression data are available from Gene Expression Omnibus (GEO) under the accession GSE107871.

## Electronic supplementary material

Supplementary Figures
