# Supplementary material for: RNA-seq identifies a diminished differentiation gene signature in primary monolayer keratinocytes grown from lesional and uninvolved psoriatic skin
Source: Sci Rep. 2017 Dec 22;7:18045. doi: 10.1038/s41598-017-18404-9 (PMC5741737; doi:10.1038/s41598-017-18404-9)

Supplemental Figures

RNA-seq identifies a diminished differentiation gene signature in primary monolayer keratinocytes grown from lesional and uninvolved psoriatic skin

William R. Swindell[1,2*], Mrinal K. Sarkar[2], Yun Liang[2], Xianying Xing[2], Jaymie Baliwag[2], James T. Elder[2], Andrew Johnston[2], Nicole L. Ward[3,4], Johann E. Gudjonsson[2]

[1]Ohio University, Heritage College of Osteopathic Medicine, Athens, OH, 45701 USA

[2]University of Michigan, Department of Dermatology, Ann Arbor, MI, 48109-2200 USA

[3]Department of Dermatology, Case Western Reserve University, 10900 Euclid Ave, Cleveland, OH, 44106, USA.

[4]The Murdough Family Center for Psoriasis, Case Western Reserve University, Cleveland, OH, USA.

[*]Corresponding Author. Email: ws277814@ohio.edu

Supplementary Figure S1. Mapping of RNA-seq reads. (A) Number of reads generated per sample. (B) Number of reads remaining after filtering of low quality reads. (C) Percentage of mapped reads. (D) Percentage of mapped reads assigned to intergenic locations. (E) Expression profiling efficiency (ratio of exon-derived reads to total reads sequenced). (F) Number of protein-coding genes with detectable expression.

Supplementary Figure S2. Exploratory analysis of RNA-seq samples. (A) Hierarchical cluster analysis. Samples were clustered based on expression values (FPKM) calculated for all protein-coding genes. (B) Hierarchical cluster analysis (adjusted expression values). Expression values were adjusted to remove the effect of subject (i.e., using residuals from linear models with subject as a covariate). (C) Principal components plot. (D) Principal components plot with two samples removed (PP Skin 3 and PN Skin 1). (E) Spearman rank correlations (skin samples). (F) Spearman rank correlations (KC samples). In (E) and (F), rank correlations were calculated between paired samples based on the expression of all protein-coding genes. (G) Variable importance. Linear models were generated for each gene with sample type (skin or KC), biopsy type (PP, PN or NN), and subject as explanatory factors. Likelihood ratio tests (LRTs) were then used to evaluate factor importance for each gene. Boxes span the middle 50% of $-\log_{10}$-transformed p-values from LRTs for each factor (whiskers: 10th – 90th percentiles).

Supplementary Figure S3. Genes with altered expression in PP-KCs compared to PN-KCs. (A, D) Top 12 genes most strongly (A) increased or (D) decreased in PP-KCs compared to PN-KCs. (B, E) MeSH terms associated with (B) PP-KC-increased genes or (E) PP-KC-decreased genes (horizontal axis: $\log_{10}$-transformed p-values, Fisher's Exact Test). (C, F) Average

expression of (C) *CALB1* and (F) *LSP1* in PP-KC, PN-KC and NN-KC samples (RNA-seq, $n = 4$ per group). (G, H) GO BP terms enriched among (G) PP-KC-increased DEGs and (H) PP-KC-decreased DEGs (horizontal axis: $\log_{10}$-transformed p-values, Fisher's Exact Test; right margin: example genes associated with each GO BP term).

Supplementary Figure S4. Genes with altered expression in PP-KCs compared to NN-KCs. (A, D) Top 12 genes most strongly (A) increased or (D) decreased in PP-KCs compared to NN-KCs. (B, E) MeSH terms associated with (B) PP-KC-increased genes or (E) PP-KC-decreased genes (horizontal axis: $\log_{10}$-transformed p-values, Fisher's Exact Test). (C, F) Average expression of (C) *KYNU* and (F) *KRT77* in PP-KC, PN-KC and NN-KC samples (RNA-seq, $n = 4$ per group). (G, H) GO BP terms enriched among (G) PP-KC-increased DEGs and (H) PP-KC-decreased DEGs (horizontal axis: $\log_{10}$-transformed p-values, Fisher's Exact Test; right margin: example genes associated with each GO BP term).

Supplementary Figure S5. Genes with altered expression in PN-KCs compared to NN-KCs. (A, D) Top 12 genes most strongly (A) increased or (D) decreased in PN-KCs compared to NN-KCs. (B, E) MeSH terms associated with (B) PN-KC-increased genes or (E) PN-KC-decreased genes (horizontal axis: $\log_{10}$-transformed p-values, Fisher's Exact Test). (C, F) Average expression of (C) *CPVL* and (F) *SPTSSB* in PP-KC, PN-KC and NN-KC samples (RNA-seq, $n = 4$ per group). (G, H) GO BP terms enriched among (G) PN-KC-increased DEGs and (H) PN-KC-decreased DEGs (horizontal axis: $\log_{10}$-transformed p-values, Fisher's Exact Test; right margin: example genes associated with each GO BP term).

Supplementary Figure S6. Expression of hyperprolferation markers. (A) Average FPKM for keratin 72 (*KRT72*/*KRT6*). (B) Average FPKM for keratin 16 (*KRT16*).

Supplementary Figure S7. Expression of genes encoding cytokines and chemokines (PP, PN and NN samples). (A) FPKM and FC estimates for genes encoding cytokines (gene symbols with "IL" prefix). (B) FPKM and FC estimates for genes encoding TNF/IFN family proteins and chemokines (gene symbols with "TNF", "IFN", "CXC" or "IFN" prefixes). In (A) and (B), genes were identified based upon indicated prefixes and those genes most strongly altered in differential expression analyses are shown. (C – G) Average FPKM for *IL36G*, *IL36RN*, *IL20RA*, *IL22RA1* and *TNFAIP8L3*. (H) Cytokine treatments most strongly altering PN-KC-decreased DEGs in a consistent direction (red: increased; blue: decreased; FDR < 0.05 for all experiments, Wilcoxon rank sum test). All experiments were performed using normal human epidermal keratinocytes in monolayer cultures unless otherwise indicated (** = reconstituted epidermis). (I) Cumulative overlap of PN-KC-decreased DEGs with genes ranked based upon their response to IL17A (GSE53751). Area indicates the size of the grey region and is the enrichment statistic shown for each cytokine experiment in (H) (p-value: Wilcoxon rank sum test).

Supplementary Figure S8. Motifs recognized by AP-1 are enriched in regions upstream of genes with decreased expression in PN-KCs. (A) Cluster analysis of 277 motifs enriched in TSS-proximal regions of PN-KC-decreased genes (FDR < 0.10). Motifs were clustered using *k*-mer scores leading to the identification of motif groups with partial preference for 5-CAG/CTG-3 and 5-ACTC/GAGT-3 elements, respectively (yellow-black heatmap). Enrichment scores quantify the degree to which motifs resemble archetypes for different DNA-binding domain

superfamily and class groups (red-black heatmaps). (B – D) TF superfamily, class and family groups most frequently associated with the 277 motifs (right margin: Fisher’s Exact Test p-value). (E) Motifs most strongly enriched among PN-KC-decreased genes (filtered to include only motifs recognized by a TF with altered expression in PN-KCs, FDR < 0.10). (F) Genes encoding Jun- and Fos-related TFs and their expression change in PN-KCs versus NN-KCs.

Supplementary Figure S9. AP-1 motifs (Fra-2|TGACTCA|M03310) in regions upstream of PN-KC-decreased genes. (A) PN-KC-decreased DEGs with the largest numbers of upstream AP-1 binding sites (5000 BP upstream from TSS). (B) AP-1 site locations for PN-KC-decreased DEGs associated with epidermal differentiation. (C) Location of AP-1 binding sites within the involucrin (*IVL*) upstream region. In (B) and (C), conserved elements correspond to regions with a phastcons score greater than 0.50. DNase I hypersensitive and FAIRE-seq sites were obtained from UCSC ENCODE annotations generated from NHEK experiments.

Supplementary Figure S1

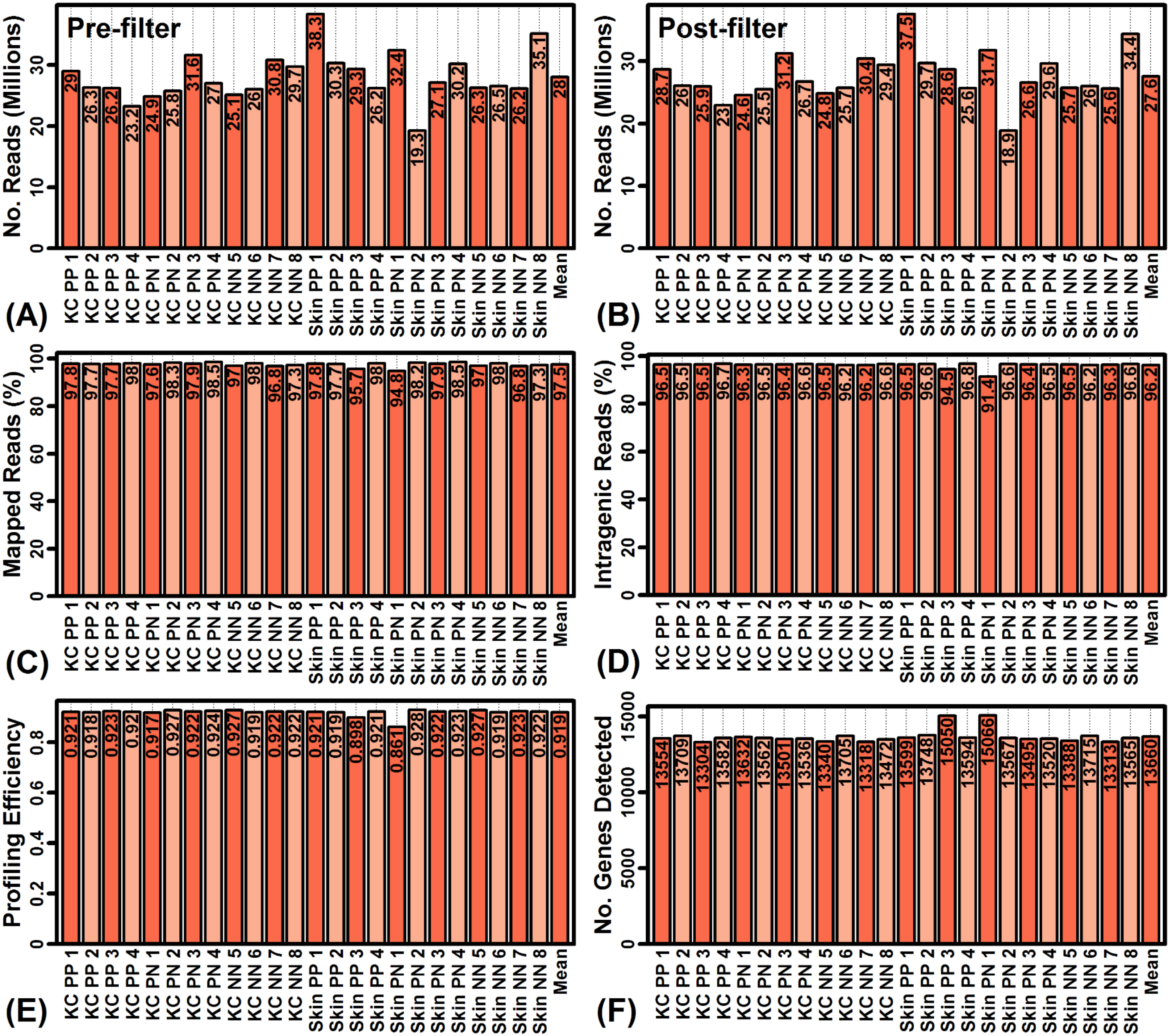

Supplementary Figure S2

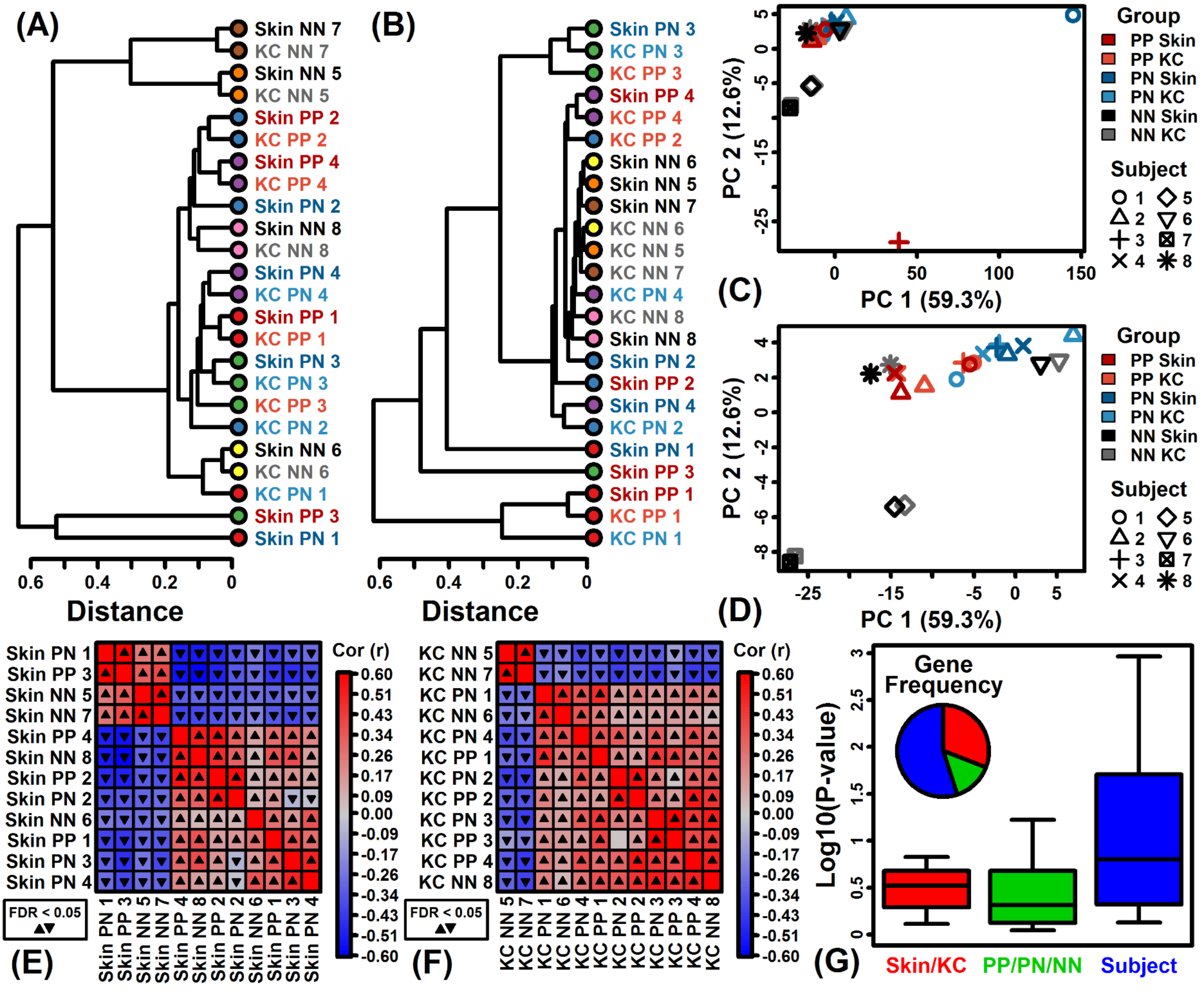

Supplementary Figure S3

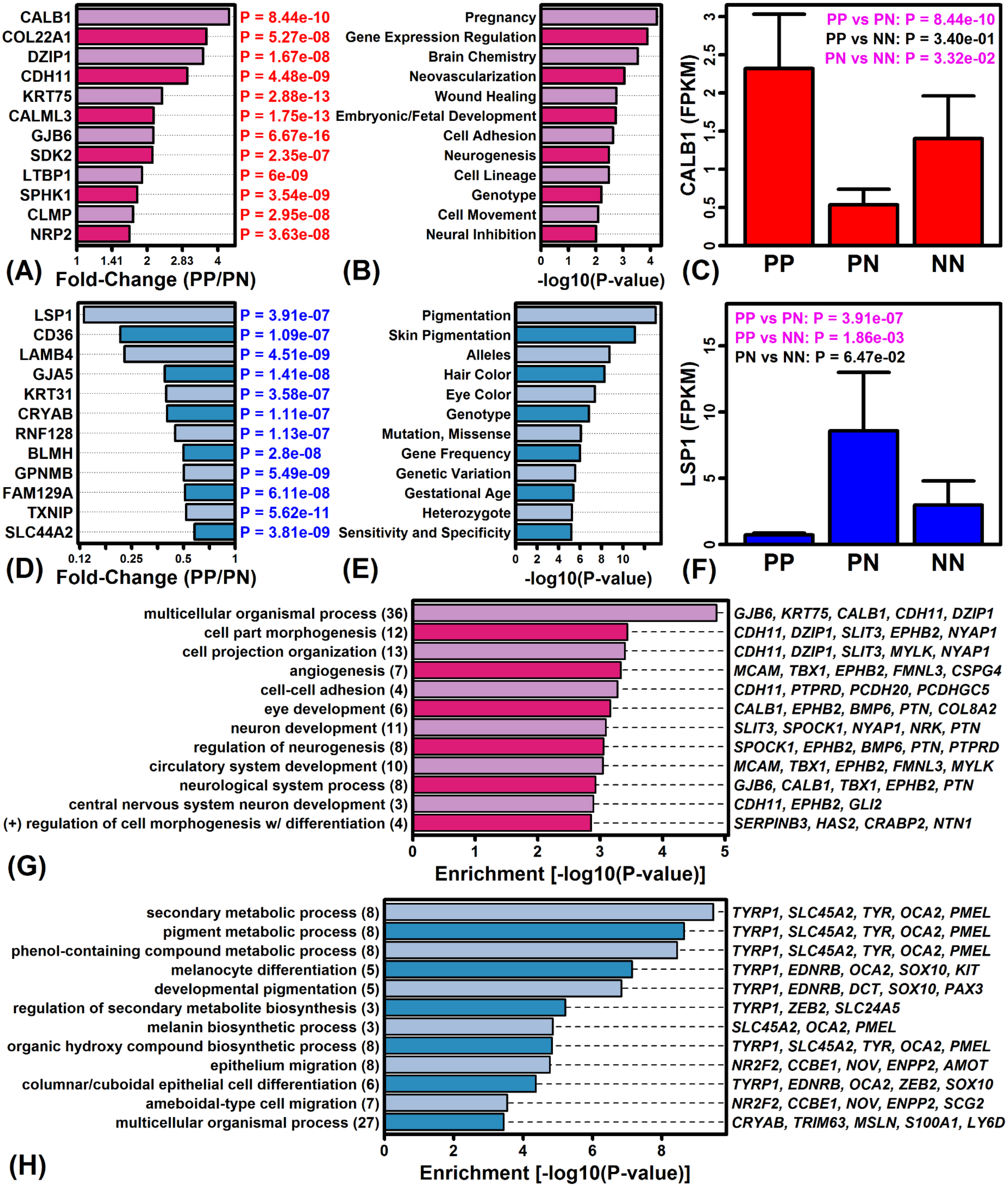

Supplementary Figure S4

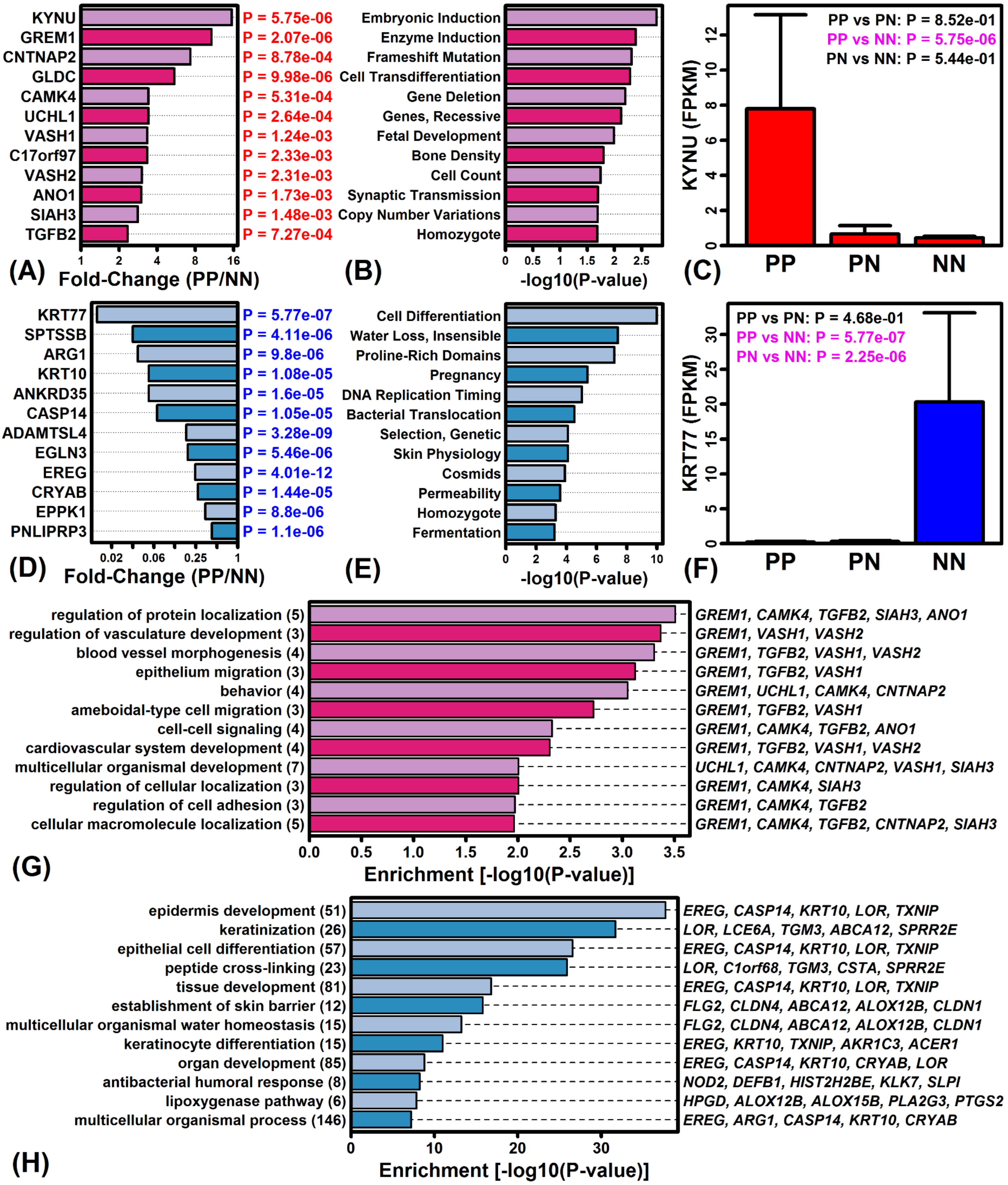

Supplementary Figure S5

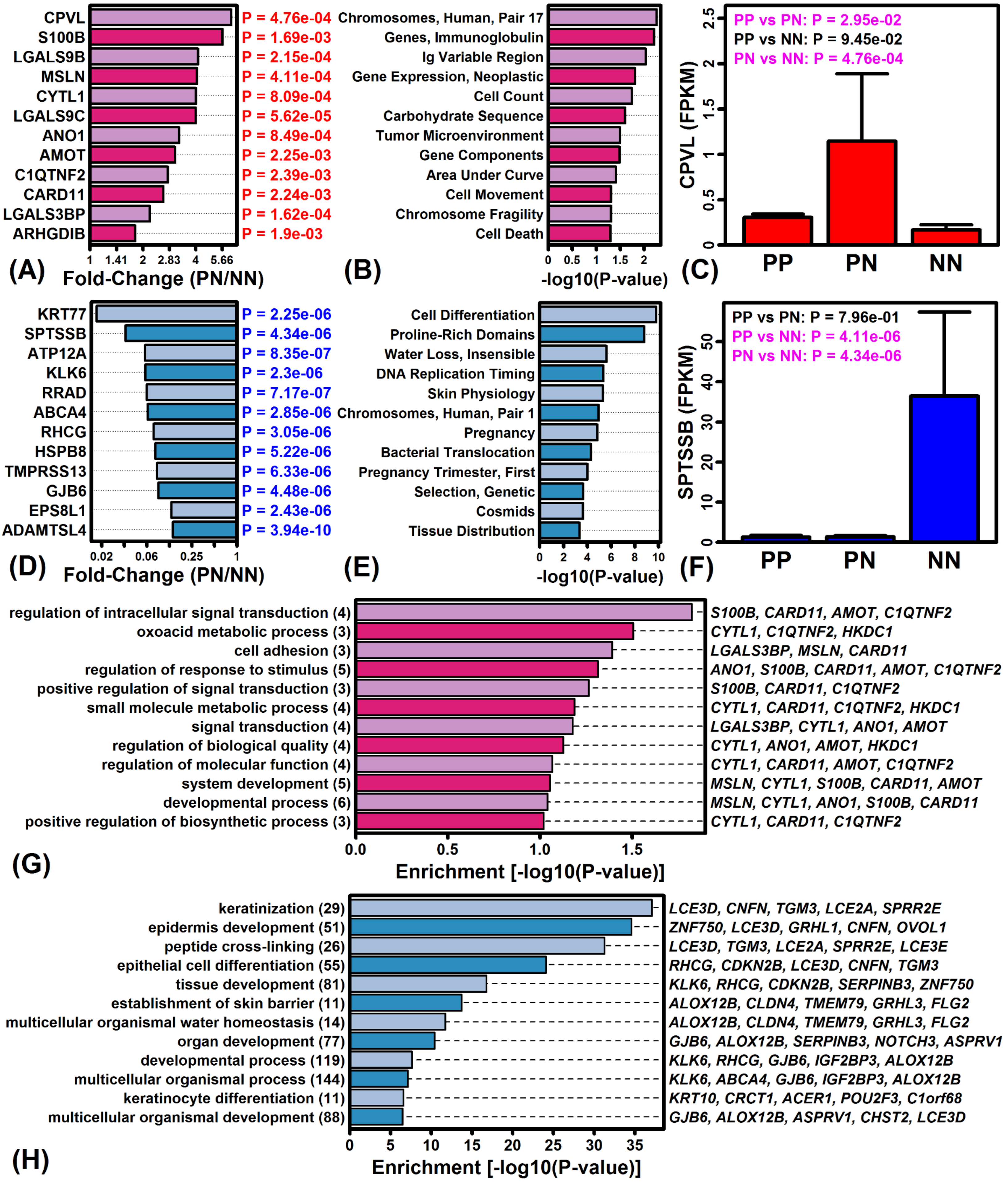

Supplementary Figure S6

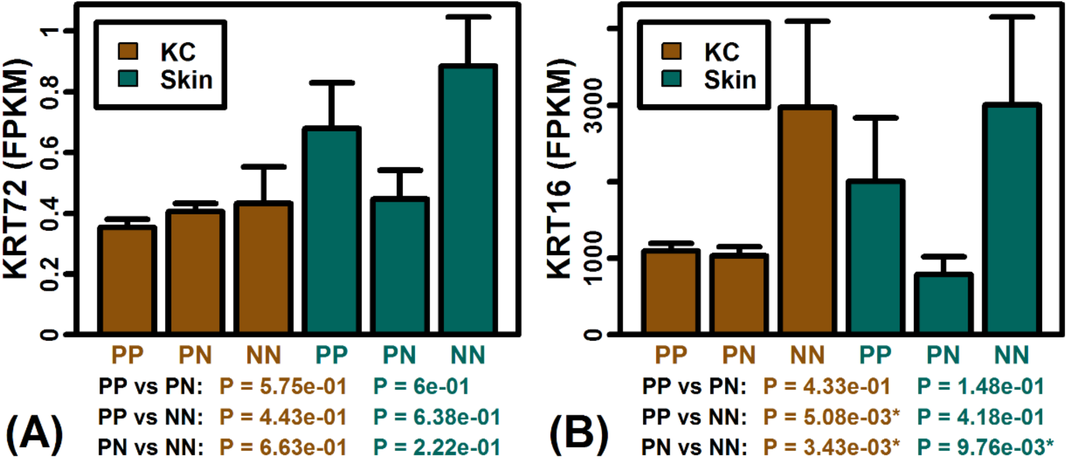

Supplementary Figure S7

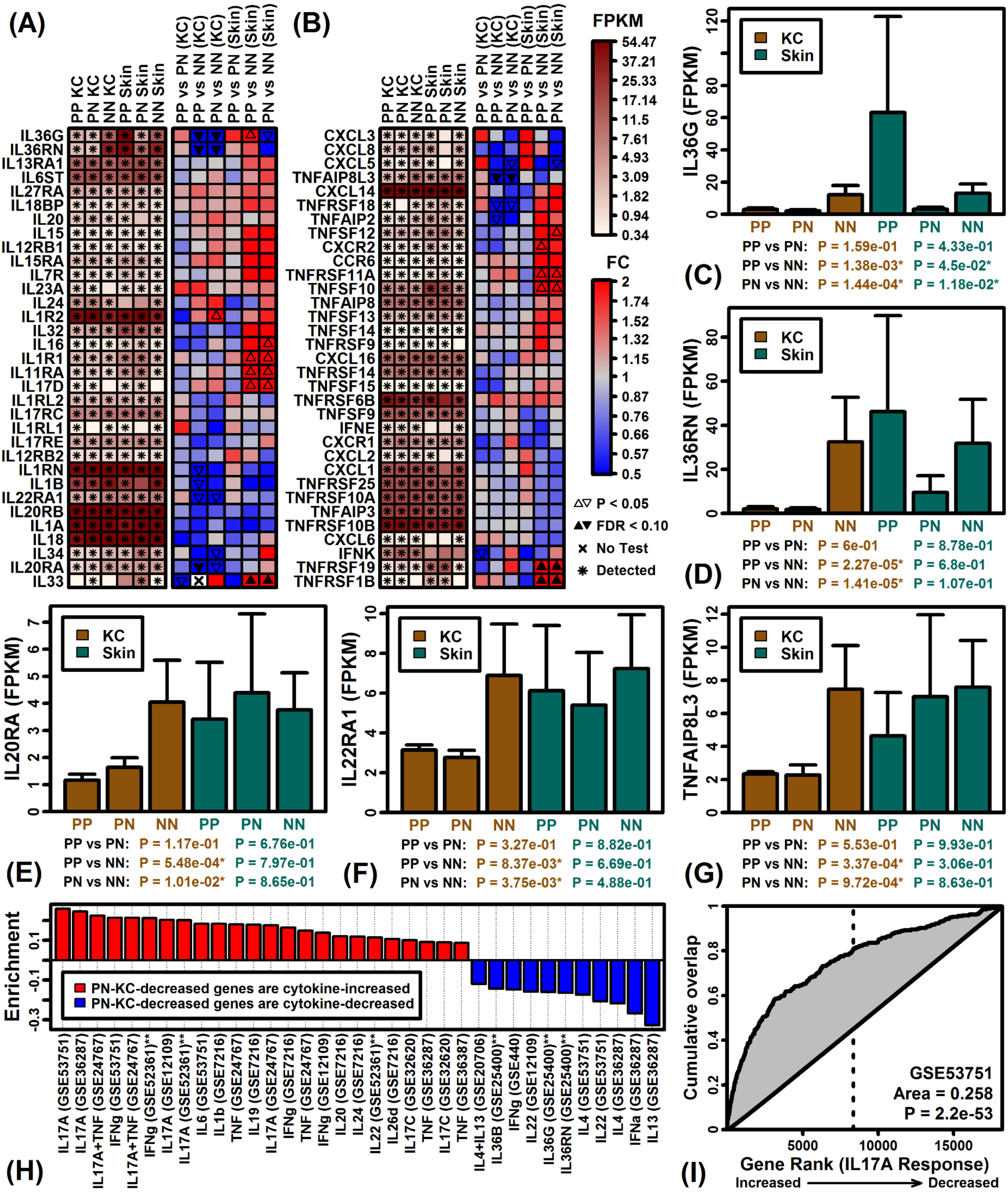

Supplementary Figure S8

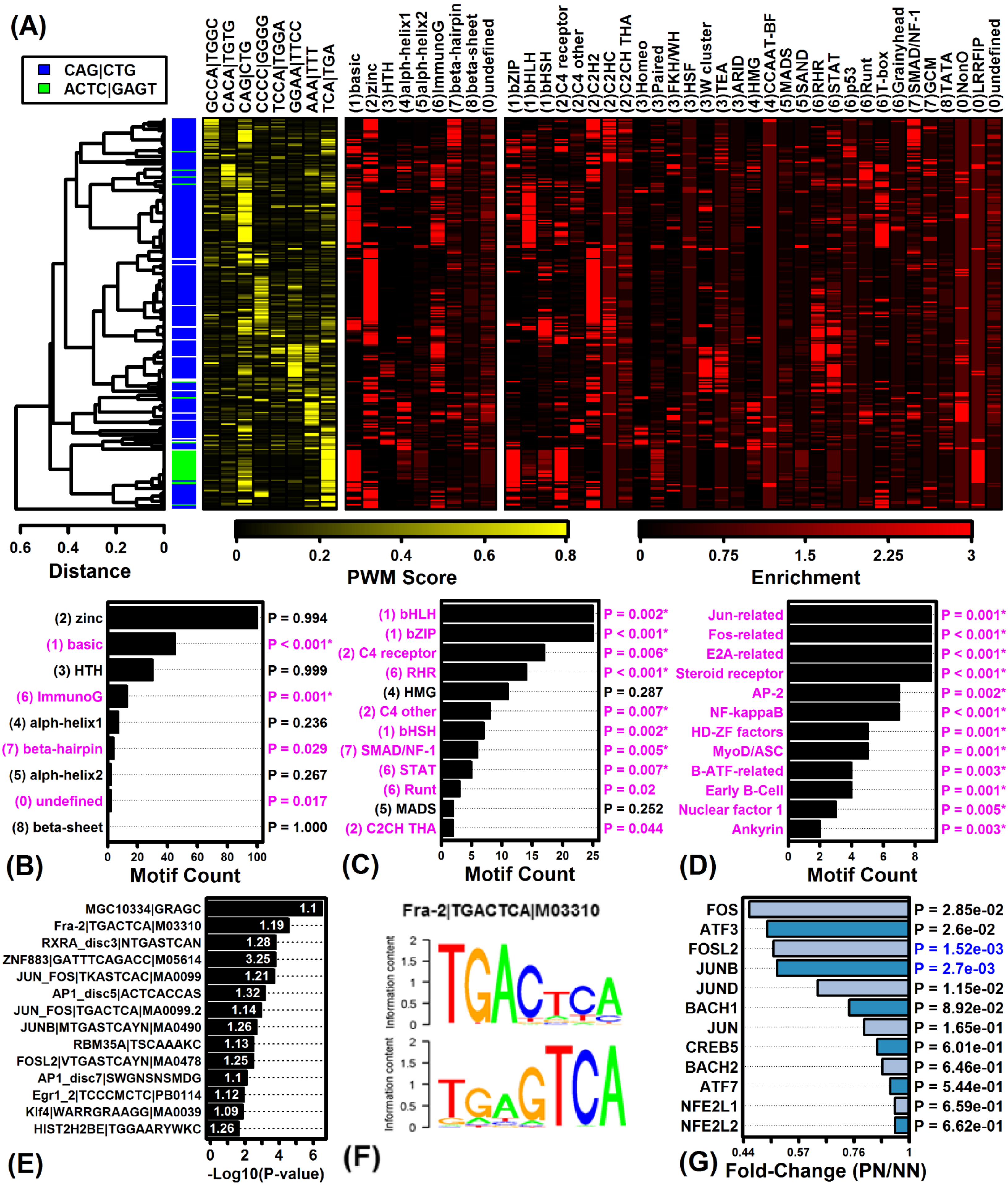

Supplementary Figure S9

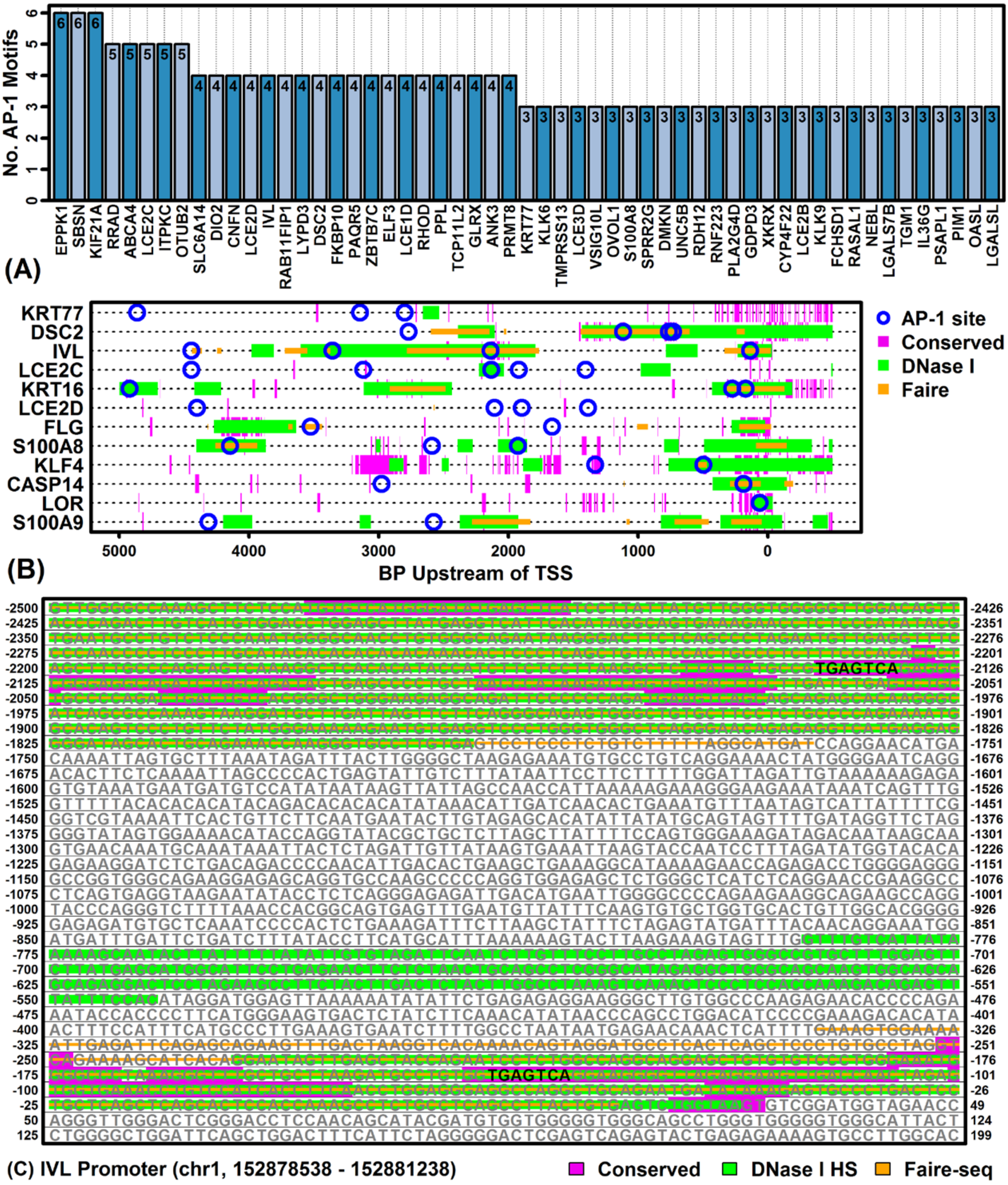

Supplement: Supplementary file 1 — Supplementary Figures [file 41598_2017_18404_MOESM1_ESM.pdf]
